# Supplementary material for: Deterministic control of ferroelectric polarization by ultrafast laser pulses
Source: Nat Commun. 2022 May 10;13:2566. doi: 10.1038/s41467-022-30324-5 (PMC9090784; doi:10.1038/s41467-022-30324-5)
Supplement: Supplementary file 1 — Supplementary Information [file 41467_2022_30324_MOESM1_ESM.pdf]

Supplementary Materials for: Deterministic control of ferroelectric polarization by ultrafast laser pulses

Peng Chen,[1,*] Charles Paillard,[2] Hong Jian Zhao,[1] Jorge Íñiguez,[3,4] and L. Bellaiche[1,†]

[1]*Physics Department and Institute for Nanoscience and Engineering, University of Arkansas, Fayetteville, Arkansas 72701, USA*
[2]*Laboratoire Structures, Propriétés et Modélisation des Solides, CentraleSupélec, UMR CNRS 8580, Université Paris-Saclay, 91190, Gif-sur-Yvette, France*
[3]*Materials Research and Technology Department, Luxembourg Institute of Science and Technology (LIST), Avenue des Hauts-Fourneaux 5, L-4362 Esch/Alzette, Luxembourg*
[4]*Physics and Materials Science Research Unit, University of Luxembourg, 41 Rue du Brill, L-4422 Belvaux, Luxembourg*
(Dated: February 2, 2022)

In this Supplemental Material, we provide information about the soft and high-frequency auxiliary modes in section I, effective Hamiltonian method in section II, discuss on the polarization trajectory in the partial- and full-excitation simulations in section III, numerically examine the essential role of the Q mode on the squeezing effect in section IV, and at last we report the strategy of polarization full-reversal under four different magnitude of laser pulse in section V.

I. FERROELECTRIC SOFT AND HIGH-FREQUENCY AUXILIARY MODES

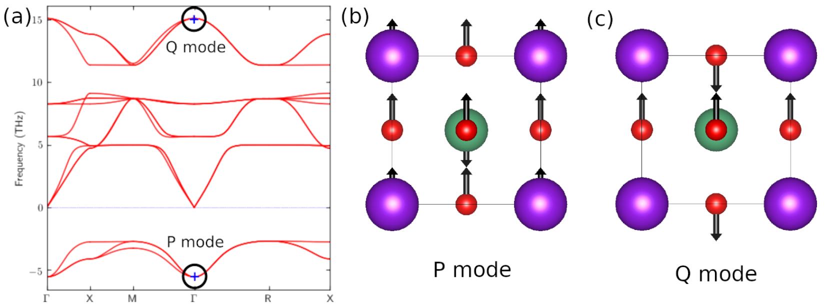

FIG. 1. (a) phonon spectra of cubic $KNbO_3$ from DFT calculations. The circled branches from top to bottom are Q and P modes; The plus sign is to indicate the modes are at Γ point. Atomic patterns corresponding to the (b) ferroelectric soft mode **P** and (c) high-frequency auxiliary mode **Q**; The color codes in (b) and (c) are as follows: red for Oxygen, green for Niobium, and purple for Potassium.

In $KNbO_3$ and other polarizable perovskites, the zone-center **P** mode is soft (in the sense that the square of its frequency is negative in the cubic phase at 0K) while the **Q** mode possesses a high and positive frequency in the cubic state at 0K. Both the distortion modes of **P** and **Q** are depicted within the perovskite 5-atom cell in figs. 1(b) and (c), respectively. Their frequencies, as calculated for $KNbO_3$ from Density Functional Theory (DFT) at 0K in the cubic phase, are marked in fig. 1(a) at the Γ point. Their eigenvalue and eigenvector are indicated in Table I. Their own

TABLE I. DFT calculated **P** and **Q** mode frequencies and eigenvectors in cubic $KNbO_3$. Note that the displacements for all the atoms are along the same direction, either x-, y- or z-direction.

	ω (THz)	K (Å)	Nb (Å)	O1 (Å)	O2 (Å)	O3 (Å)
Q mode	15	0.010	-0.056	-0.773	0.447	0.447
P mode	-5	0.053	-0.654	0.506	0.396	0.396

* peng.chen.iphy@gmail.com
† laurent@uark.edu

dielectric responses at finite temperatures (and thus in different phases) are shown in fig. 2, as computed from the effective Hamiltonian simulations following the formalism in [1, 2]. Focusing on the dielectric response associated with $\mathbf{Q}$, its peaks correspond to the resonance of the high-frequency auxiliary modes. One can see that, as consistent with experiments [3, 4], such resonance for A and E modes are about 18 and 16 THz respectively in the R-phase at 240K (figs. 2(a)), for A, B1 and B2 modes are 18, 16, and 15.5 THz respectively in the O-phase at 300K (figs. 2(b)), for A and E modes are 19 and 15.5 THz respectively in the T-phase at 400K (figs. 2(c)), and T mode (which is also the F mode [3, 4]) is 16 THz in the C-phase at 800 K (figs. 2(d)). One can also see that the calculated peaks for resonant frequencies are in good agreement with the experimental data of Refs. [3, 4] (marked by vertical lines in figs. 2) of rhombohedral and orthorhombic phases; The deviation of numerical results from the experiment in tetragonal and cubic phases are within 1 THz. Note that in the numerical experiments on squeezing effect, we choose the frequency to excite the A-mode of Q ($\mathbf{Q}_A$) in order to trigger the squeezing. Because A mode defines the oscillation along the polarization direction and E and B modes are perpendicular to the polarization. As we are going to discuss in Section III of this Supplemental Material, the existence of A and E modes in the R-phase can affect the polarization switching when laser pulses of 18 THz are applied in the R-phase, as documented in Fig. 2(d) of the main manuscript. Moreover, considering that the linear response to the electric field excitation, $\mathbf{E}\cdot\mathbf{Q}$, is proportional to $\frac{\mathbf{E}\cdot\mathbf{Q}}{\omega^2-\omega_i^2}$, where ω_i are the eigenfrequencies of the system, we used in the manuscript laser pulses 18 THz for R- and O-phase and 19 THz for T-phase, that is with a frequency that is close enough to to the resonance of the high-frequency auxiliary mode and far enough to the low-frequency resonance of the soft-mode (which can be found in fig. 2 by looking at the peaks of the dielectric response associated with $\mathbf{P}$, and which is thus around 8-9 THz for all three ferroelectric phases).

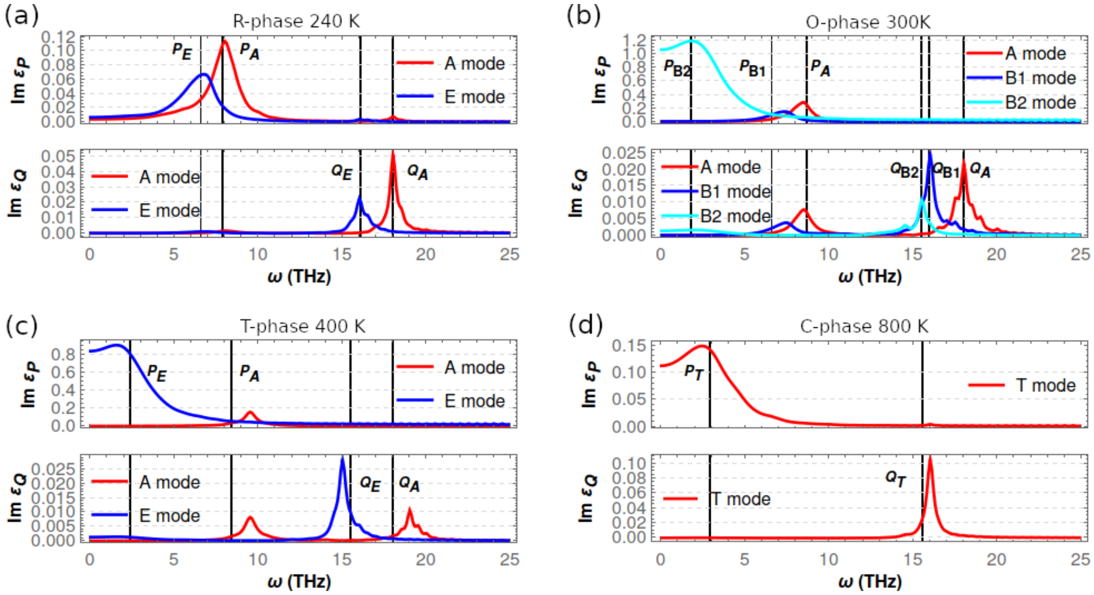

FIG. 2. Imaginary part of dielectric constant for both the $\mathbf{P}$ and $\mathbf{Q}$ modes in the (a) R-phase at 240 K; (b) O-phase at 300 K; (c) T-phase at 400 K; and (d) C-phase at 800K. The peaks for A modes correspond to oscillations parallel to the polarization while for E and B modes they correspond to oscillation being perpendicular to the polarization. The T mode in cubic is triply degenerated as there is no inversion symmetry breaking. The measured frequencies are marked by the vertical black lines in order to be compared with our numerical results.

II. EFFECTIVE HAMILTONIAN

Here, we describe in detail the newly developed effective Hamiltonian (H_{eff}) of ferroelectric polarization ($\mathbf{P}$) and high-frequency auxiliary mode ($\mathbf{Q}$) coupled system, and its coefficients for $KNbO_3$. It has the following degrees of freedom: vectors related to the ferroelectric soft mode $\mathbf{P}$, high-frequency auxiliary mode $\mathbf{Q}$ and inhomogeneous strain ($\mathbf{u}$) in each 5-atom unit cell, as well as, the homogenous strain (η). Both P and Q modes are infrared-active modes. Their associated local vectors in the H_{eff} are centered on Nb ions. The local vectors corresponding to the inhomogeneous strains are technically centered on K ions. The homogenous strain is defined with respect to cubic symmetry and has six independent components η_i, in Voigt notation.

TABLE II. Expansion parameters of the effective Hamiltonian for $KNbO_3$. Atomic units are used here. The reference cubic lattice parameter is 7.464 Bohr

Dipole	Z_p	11.1697	Z_q	-3.9183	ϵ_∞	6.649
M_p	45	M_q	16	M_u	180	
$E^{onsite}(P)$	κ_p	0.0729011	α_p	0.38937	γ_p	-0.68078
	j_1^p	-0.0130602	j_2^p	0.0061562		
$E^{nn}(P)$	j_3^p	0.0037809	j_4^p	-0.0024885	j_5^p	0.01050
	j_6^p	0.00125679	j_7^p	0.00062839		
$E^{int}(P,\eta)$	C^p_{1111}	-2.609	C^p_{1122}	0.38441	C^p_{1212}	-0.019093
$E^{elastic}$	B_{11}	6.089	B_{12}	1.124	B_{44}	1.329
$E^{onsite}(Q)$	κ_q	0.079852	α_q	0.031271	γ_q	-0.024934
	j_1^q	-0.0016375	j_2^q	0.0046928		
$E^{nn}(Q)$	j_3^q	0.0010588	j_4^q	-0.0008193	j_5^q	0.00
	j_6^q	0.00037143	j_7^q	0.00018572		
$E^{int}(Q,\eta)$	C^p_{1111}	-1.4584	C^p_{1122}	-0.22048	C^p_{1212}	0.122845
	Λ_{22}	0.47182				
$U^{int}(P,Q)$	Λ_1	-0.04958	Λ_2	-0.4849	Λ_3	-0.397024
	Λ_{211}	-0.06446	Λ_{112}	0.10272	Λ_{1111}	0.19742

The potential energy in $H_{eff} = K^{tot} + U^{tot}$ is the sum of four main energies:

$$U^{tot} = U^{FE}(\{\mathbf{P}\},\{\mathbf{u}\},\{\eta\}) + U^{aux}(\{\mathbf{Q}\},\{\mathbf{u}\},\{\eta\}) + U^{int}(\{\mathbf{P}\},\{\mathbf{Q}\}) + U^{elastic}(\{\eta\}) \tag{1}$$

where U^{FE} is the energy associated to the ferroelectric soft mode and its interaction to homogeneous and inhomogeneous strains; U^{aux} is the energy associated to the high-frequency auxiliary mode and its interaction to homogeneous and inhomogeneous strains; U^{int} includes the essential direct interactions between $\mathbf{P}$ and $\mathbf{Q}$ modes; and $U^{elastic}$ is the elastic energy.

U^{FE} contains the following five terms, as proposed in Ref.[5]

$$\begin{aligned} U^{FE} &= E^{onsite}(\{\mathbf{P}\}) + E^{dpl}(\{\mathbf{P}\}) \\ &+ E^{nn}(\{\mathbf{P}\}) + E^{int}(\{\mathbf{P}\},\{\mathbf{u}\},\{\eta\}) \end{aligned} \tag{2}$$

where E^{onsite} is the $\mathbf{P}$ mode on-site interaction within a unit cell, E^{dpl} is the long range dipole-dipole interaction, E^{nn} represents the short range interactions between neighboring $\mathbf{P}$ modes excluding dipole-dipole interactions, and E^{int} is the interaction between elastic deformation and $\mathbf{P}$ modes. Their specific expressions read as follows:

$$E^{onsite} = \sum_i^N \{\kappa_p \mathbf{P}_i^2 + \alpha_p \mathbf{P}_i^4 + \gamma_p (P_{ix}^2 P_{iy}^2 + P_{iy}^2 P_{iz}^2 + P_{iz}^2 P_{ix}^2)\} \tag{3}$$

$$E^{dpl} = \frac{1}{2}\frac{Z_p^2}{\epsilon_\infty}\sum_{i\neq j}^N \frac{\mathbf{P}_i\cdot\mathbf{P}_j - 3(\mathbf{d}_{ij}\cdot\mathbf{P}_i)(\mathbf{d}_{ij}\cdot\mathbf{P}_j)}{d_{ij}^3} \tag{4}$$

$$E^{nn} = \frac{1}{2}\sum_{i\neq j}^N J^p_{ij\alpha\beta} P_{i\alpha} P_{j\beta} \tag{5}$$

$$E^{int} = \sum_i^N \sum_{\alpha\beta\mu\nu}^{x,y,z} C^p_{\alpha\beta\mu\nu}\varepsilon_{\alpha\beta} P_{i\mu} P_{i\nu} \tag{6}$$

where the sums on i and j run over N unit cells, α, β, μ, and ν are Cartesian components along the x, y, and z axes, and $\mathbf{d}_{ij}$ represents the vector that points from site i to site j. Note that strains $\varepsilon_{\alpha\beta}$ contain both homogeneous strains $\{\eta\}$ and inhomogeneous-strain related variables $\{\mathbf{u}\}$. The site-site interaction tensor $J_{ij\alpha\beta}$ in E^{nn} can be simplified for different nearest neighbor (NN) shells as:

$$J_{NN^{1st}\alpha\beta} = (j_1 + (j_2 - j_1) d_{ij,\alpha})\delta_{\alpha\beta} \tag{7}$$

$$J_{NN^{2nd}\alpha\beta} = (j_4 + \sqrt{2}(j_3 - j_4) d_{ij,\alpha})\delta_{\alpha\beta} + 2 j_5 \mathbf{d}_{ij,\alpha}\cdot\mathbf{d}_{ij,\beta}(1-\delta_{\alpha\beta}) \tag{8}$$

$$J_{NN^{3rd}\alpha\beta} = j_6\delta_{\alpha\beta} + 3 j_7 \mathbf{d}_{ij,\alpha}\cdot\mathbf{d}_{ij,\beta}(1-\delta_{\alpha\beta}) \tag{9}$$

The potential energy U^{aux} follows the same formalism as U^{FE} only with different parameters, to be denoted as κ_q, α_q, γ_q, Z_q, J^q, and C^q.

Moreover, the interaction between $\mathbf{P}$ and $\mathbf{Q}$ is written as:

$$
\begin{aligned}
U^{int} = & \sum_i^N \sum_\alpha^{x,y,z} \sum_{n=1}^3 \Lambda_n P_{i\alpha}^n Q_{i\alpha}^{4-n} \\
& + \sum_i^N \Lambda_{1111}(P_{ix}P_{iy}Q_{ix}Q_{iy} + P_{iy}P_{iz}Q_{iy}Q_{iz} + P_{iz}P_{ix}Q_{iz}Q_{ix}) \\
& + \sum_i^N \Lambda_{22}(P_{ix}^2(Q_{iy}^2 + Q_{iz}^2) + P_{iy}^2(Q_{iz}^2 + Q_{ix}^2) + P_{iz}^2(Q_{ix}^2 + Q_{iy}^2)) \\
& + \sum_i^N \Lambda_{211}(P_{ix}^2(P_{iy}Q_{iy} + P_{iz}Q_{iz}) + P_{iy}^2(P_{iz}Q_{iz} + P_{ix}Q_{ix}) + P_{iz}^2(P_{ix}Q_{ix} + P_{iy}Q_{iy})) \\
& + \sum_i^N \Lambda_{112}((P_{iy}Q_{iy} + P_{iz}Q_{iz})Q_{ix}^2 + (P_{iz}Q_{iz} + P_{ix}Q_{ix})Q_{iy}^2 + (P_{ix}Q_{ix} + P_{iy}Q_{iy})Q_{iz}^2)
\end{aligned}
\tag{10}
$$

where n is the power order that goes from 1 to 3, i is the unit cell index that goes through all N sites. The elastic energy has the forms of

$$
U^{elastic} = \frac{N}{2} \sum_{\alpha\beta\mu\nu}^{x,y,z} B_{\alpha\beta\mu\nu} \varepsilon_{\alpha\beta} \varepsilon_{\mu\nu} \tag{11}
$$

where the strain tensor ε includes both homogeneous ($\{\eta\}$) and inhomogeneous ($\{\mathbf{u}\}$) strains. The parameters for the potential energy are first fitted from density functional theory (DFT) total energy and Hessian calculations, but then some of them are modified as indicated in the main manuscript. They are listed in Table II in atomic units.

Moreover, the kinetic energy K^{tot} of the H_{eff} contains three parts written with respect to the velocity $\mathbf{v}_p$, $\mathbf{v}_q$, and $\mathbf{v}_u$ that correspond to the order parameters, $\mathbf{P}$, $\mathbf{Q}$, and $\mathbf{u}$:

$$
K^{tot} = \sum_i^N \frac{1}{2} M_p v_{p,i}^2 + \frac{1}{2} M_q v_{q,i}^2 + \frac{1}{2} M_u v_{u,i}^2 \tag{12}
$$

where the effective masses are defined as M_p, M_q, and M_u respectively. Their values are fitted to produce the same mode frequency as from the DFT phonon calculations at 0 K, and are listed in Table II too. Using these parameters, we employed Monte Carlo (MC) and Molecular Dynamic (MD) algorithms on a 12×12×12 supercell that contains 8640 atoms. More specifically, we used parallel tempering[6,7] (PT) MC and Nosé-Hoover thermal state[8–10] MD simulations implemented in LINVARIANT[11]. Periodic boundary conditions were adapted. For each temperature, 150,000 PTMC sweeps are firstly performed, with the first 100,000 steps as thermalization and the subsequent 50,000 steps to compute the phase diagram; then MD simulations are initialized with the MC outputs and 500,000 thermalization steps are performed before the statistical evaluations; the time interval of 0.1fs is used in the MD simulations.

III. PARTIAL- AND FULL-EXCITATION

In this section and in order to complement results of "Electrical polarization reversal" section of the main manuscript, the trajectories of each components of $\mathbf{P}$ and $\mathbf{Q}$ modes at 240K under laser pulse are summarized. We used a Gaussian-enveloped sinusoidal electric field to mimic the laser pulse in the experiment[12]. The halfwidth is set to be 200fs and the frequency is chosen at 18 THz which is close enough to the Q mode frequency in the R-phase. Different magnitude of electric fields that are parallel to the initial electrical polarization direction (that is $[11\bar{1}]$) are used and marked in figs. 3 and 4.

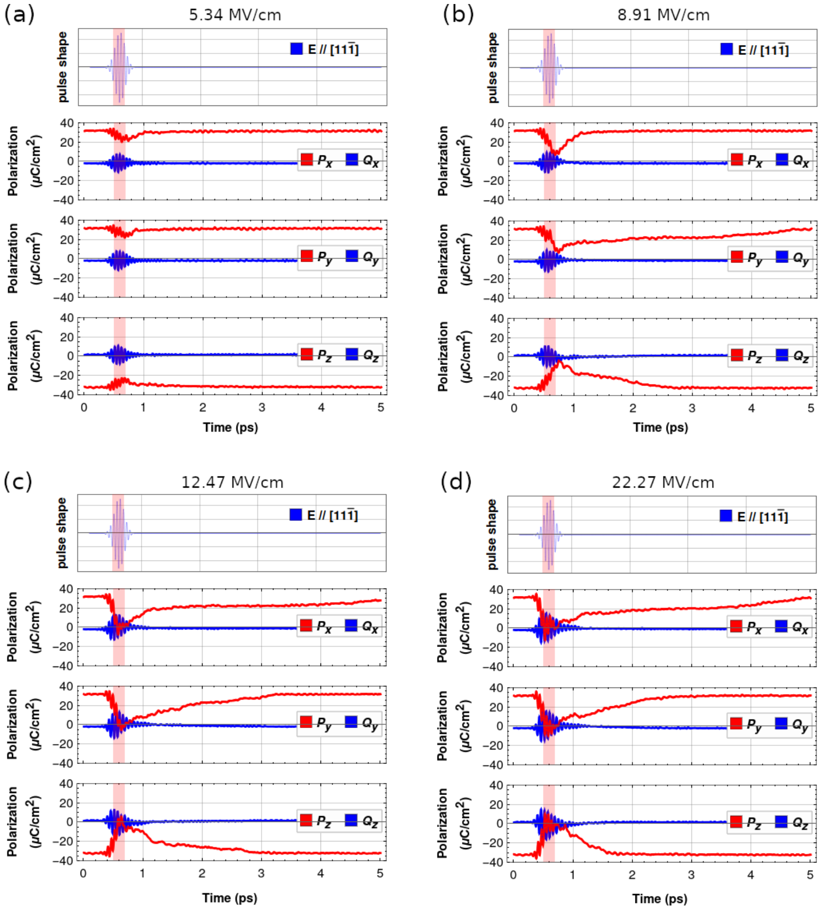

FIG. 3. partial-excitation associated with Fig 1(c) of the main text; the pink regions are used to indicate the full-width-half-maximum of the pulse.

In the case of partial-excitation: We can see that Q_x, Q_y, and Q_z are oscillating with large amplitudes, and, consequently (because of squeezing effects), all P_x, P_y, and P_z decrease in magnitude wiggly during this period (when the pulse has large electric field) in the four cases of fig. 3. The difference among them is the dip that the polarization can reach with the kinetic energy gained from different magnitude of electric fields when the pulse starts to get reduced. More specifically, the P_x, P_y, and P_z in fig.3 (a) with E=5.34 MV/cm are such as the total polarization goes towards the [$\bar{1}\bar{1}$1] direction first and then bounces back to [11$\bar{1}$], during which period none of P_x, P_y, and P_z touches a zero value. This is because the kinetic energy that $\mathbf{P}$ modes gain during the laser pulse is not large enough to overshoot to the opposite direction. In the case of a larger electric field, E=8.91 MV/cm, in fig.3 (b), P_x, P_y, and P_z touch zero for a short moment, but none of them crosses over the zero point. So no polarization reversal happens either in this case. On the other hand, when the electric field increases to E=12.47 MV/cm, P_x, P_y, and P_z gain enough kinetic energy during the pulse and continue to go over the zero point when the pulse is almost out and when thus the squeezing effect is quite weak. Thus the reversal happens but not the full one, because, at that moment in the dip, the force from the un-excited part of the material that wants to align the polarization along [11$\bar{1}$] direction starts to overcome the squeezing effect (that wants the polarization to be zero). On the microscopic level, in the dip, some cells exposed to the laser present polarization along the [$\bar{1}\bar{1}$1] direction and other cells start to deviating toward the [11$\bar{1}$] direction. For an even larger field, such as E=22.27 MV/cm, not only P_x, P_y, and P_z gain enough kinetic energy to drop to zero, but also the squeezing effect is still large at that moment. This is because the electric field is so large that within a fixed pulse width (as τ is equal to 200 fs for all these cases) there is a longer duration for which the electric field is large enough to excite large $\mathbf{Q}$ mode and thus to provide longer duration of squeezing. This is why P_x, P_y, and P_z oscillate several times near zero before recovering their initial value.

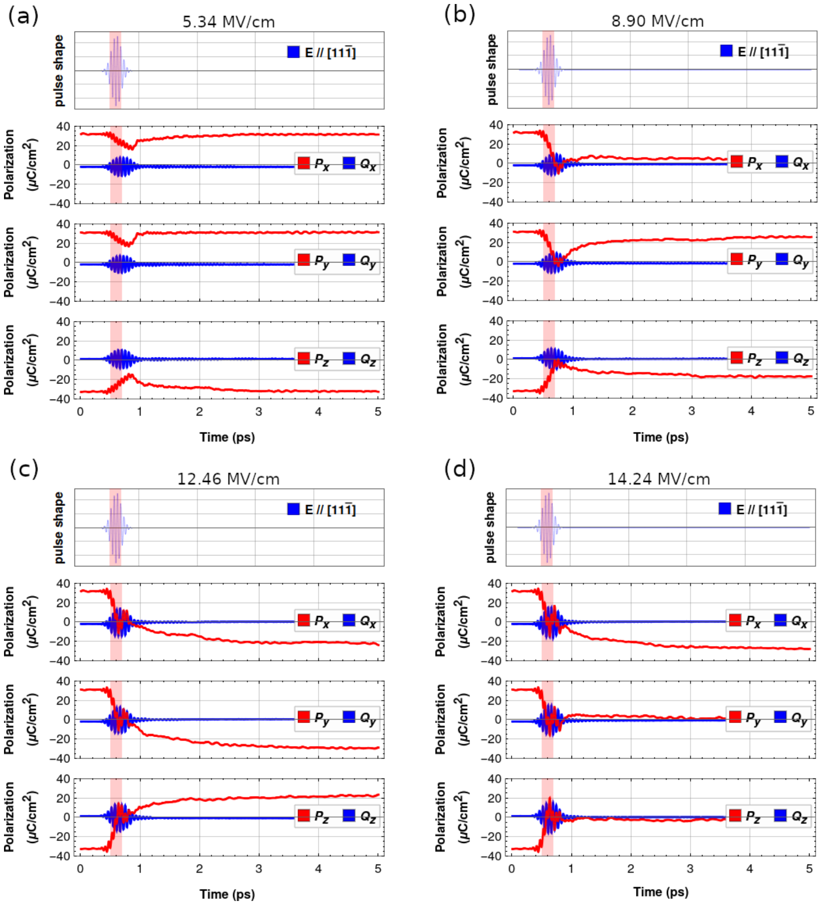

FIG. 4. full-excitation associated with Fig 1(d) of the main text; the pink regions are used to indicate the full-width-half-maximum of the pulse.

In the case of full-excitation: We can also see that P_x, P_y, and P_z decrease wiggly as a reaction to any considered pulse, as in the partial-excitation cases and as consistent with squeezing effects. In fig. 4(a) and for the small field of 5.34 MV/cm, the behavior of P_x, P_y, and P_z is exactly like in the case of partial-excitation with E=5.34 MV/cm as depicted in fig. 3(a): after experiencing a short moment of decreasing but with no change in their sign, they recover their initial value after the pulse exits the system. For a larger electric field of E=8.90 MV/cm, in fig. 4(b), P_x, P_y and P_z touch zero. When the pulse is out, only P_y and P_z start to recover toward their initial value, but P_x stays near zero. The microscopic origin of this vanishing of P_c is found to be the coexistence of two R-domains, one for which the polarization is along the initial $[11\bar{1}]$ direction and another one for which the polarization has rotated to be along $[\bar{1}1\bar{1}]$ via a change of sign of P_x. Interestingly, the coexistence of domains when under such type of laser pulses was also experimentally observed in $SrTiO_3$[13]. Moreover, rotation to other R-phases also happen in fig.4 (d) for a field of 14.24 MV/cm, including domains along $[\bar{1}1\bar{1}]$, $[\bar{1}\bar{1}\bar{1}]$, $[\bar{1}11]$, and $[\bar{1}\bar{1}1]$ directions. In the case of fig. 4(c) with E=12.46 MV/cm, the three polarization components change their signs and thus the polarization is fully reversed to $[\bar{1}\bar{1}1]$. Note that, in these two cases of 12.46 and 14.24 MV/cm, when P_x, P_y, and P_z touch zero, the pulse is around its maximum. So oscillation of polarization's components near zero due to squeezing can be observed. After these oscillations around zero, equal volume of positive and negative domains (e.g. polarization component adopts either positive or negative values) happen along the y and z-axes for the largest field of 14.24 MV/cm (see fig. 4(d)), which explains why P_y and P_z are all zero. The whole fig. 4 therefore shows that not only reversal of the polarization, but also rotation to other R-phases as well as formation of R-domains, are also possible when activating the $\mathbf{Q}$ mode by a pulse. One needs two conditions to be fulfilled in order to end up with a full reversal of the polarization: the first one is that the pulse should end at the exact moment when all polarization components touches zero, while the second one is that the polarization should have gained enough kinetic energy to go over the zero point when the pulse is out. Without the first condition, the polarization will be squeezed to zero with orientation and magnitude fluctuations and the system can then adopt other R-phases with rotating polarization when the electric field is totally out. Furthermore, if the second condition can not be matched, P_x, P_y and P_z will not have enough kinetic energy to go over the zero point (where the highest energy locates), and the polarization can then recover its initial value.

Another interesting way to understand the different results of fig. 4 is that the application of 18 THz pulses can activate the A-mode of **Q** but also its E-mode, since both of these two modes are close to each other in frequency in the R-phase (see Section I of this Supplemental Material). The activation of this E-mode can then facilitate the rotation of the polarization (as consistent with the results of fig. 4 for fields of 8.90 and 14.24 MV/cm) while activating the A-mode of **Q** can induce a reversal of the polarization (as consistent with the results of fig. 4 for the field of 12.46 MV/cm).

IV. ROLE OF P AND Q ON THE SQUEEZING EFFECT

Let us concentrate on a T-phase with an initial **P** along [001] and apply a laser pulse E=7.71 MV/cm parallel to [001] direction as in fig. 2 of the main text. Here, we keep all the parameters and initial conditions identical to the one in Fig. 2 of the main text except that the **Q** mode is not allowed to be oscillating (its velocity is fixed to zero) and thus frozen in its (negative) equilibrium value in such initial T-phase. The response of **P** mode in such conditions can be seen in fig. 5(c): P_z "only" oscillates around its equilibrium value rather than decrease to zero (as in Fig. 2 of the main text). Such numerical experiment demonstrate that the response of **Q** to a pulse is needed in order to induce a squeezing effect on **P**.

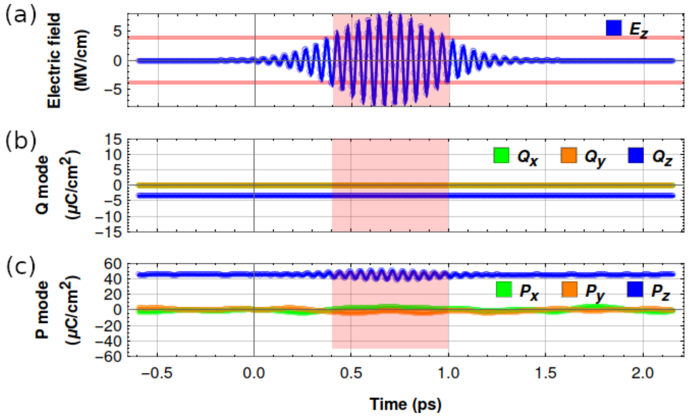

FIG. 5. Numerical experiment in which the set up is the same as in Fig. 2 of the main text, except that **Q** mode is frozen in its equilibrium value

Note that other numerical experiments also revealed that, in addition to the $U_{22}^{(z)}$ and $U_{22}^{(x-z)}$ as discussed in the main text, the roles of U_{31} and U_{13} as defined in eq. 13 and 14 are also important in the squeezing effect both quantitatively and qualitatively.

$$U_{31} = \Lambda_3(P_x^3 Q_x + P_y^3 Q_y + P_z^3 Q_z) \tag{13}$$

$$U_{13} = \Lambda_1(P_x Q_x^3 + P_y Q_y^3 + P_z Q_z^3) \tag{14}$$

As a matter of fact, the significant coefficients Λ_1 and Λ_3 in the front of U_{13} and U_{31} result in couplings that pump energy efficiently from the resonated Q modes into P modes, and thus make the squeezing effect happens within sub-picoseconds. More precisely, the larger the coefficients in front of U_{13} and/or U_{31} the smaller Q amplitude (and thus smaller pulse) is needed to give rise to efficient squeezing effect, which also means a lower demand on the laser power. Moreover, the coefficients in front of U_{31} and U_{13} play another important role in $KNbO_3$: not only do they drive the condensation of Q_z when P_z exists (Q_z would be zero if such couplings did not exist) but their positive signs also force Q_z and P_z to be of opposite signs – as evidenced in figs. 1(a,b) and figs. 2(b,c) of the main text.

V. STRATEGY OF FERROELECTRIC POLARIZATION FULL-REVERSAL

In fig. 6, we numerically check the strategy proposed in the Section "Ultrafast full-reversal strategy" of the main text and summarized in fig. 3(a) of the main text, using different magnitude of laser pulses, namely 5.14, 6.68, 7.71, and 9.26 MV/cm, to realize deterministic polarization full reversal. A *dc* gate field is constantly applied along the $[\bar{1}10]$ direction, with its amplitude equal to 0.0154 MV/cm. The sequence of laser pulse includes a x-polarized pulse at 10 ps, a y-polarized pulse at 20 ps, and a z-polarized pulse at 30 ps, as detailed in the main text. The initial state

is an O-phase with a polarization along the $[1\bar{1}0]$ direction. The process in figs. 6 (a) and (d) goes through the same transition path as the one described in the main text, that is ①③⑤. Furthermore panels (b) and (c) show the other predicted path, namely ②④⑤. Both transition paths do end up with a full reversed polarization of the polarization from the initial $[1\bar{1}0]$ direction to the final $[\bar{1}10]$ direction.

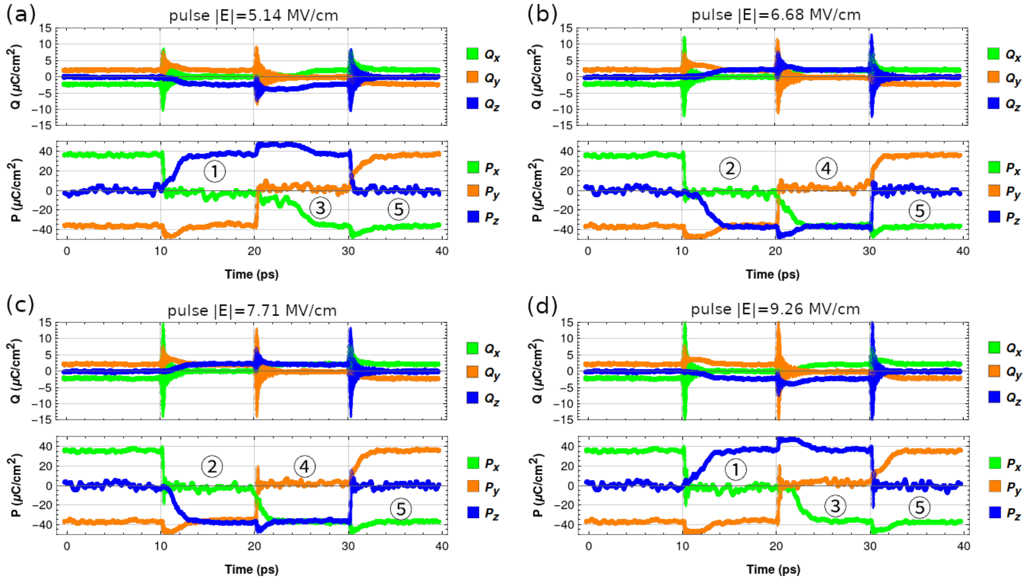

FIG. 6. Strategy for realizing the full-reversal of the ferroelectric polarization with different magnitude of laser pulse, e.g. 5.14, 6.68, 7.71, and 9.26 MV/cm. The gate field is along $[\bar{1}10]$ with a magnitude of E=0.0154 MV/cm. The circled digits are the same markers indicated in fig. 3 (a) of the main text.

---

1. Caillol, J. M., Levesque, D. & Weis, J. J. Theoretical calculation of ionic solution properties. The Journal of Chemical Physics **85**, 6645–6657 (1986).
2. Wang, D., Weerasinghe, J., Bellaiche, L. & Hlinka, J. Dynamical coupling inPb(zr, ti)o3solid solutions from first principles. Phys. Rev. B **83** (2011).
3. Fontana, M. D., Kugel, G. E., Metrat, G. & Carabatos, C. Long-wavelength phonons in the different phases of KNbO3. physica status solidi (b) **103**, 211–219 (1981).
4. Fontana, M. D., Metrat, G., Servoin, J. L. & Gervais, F. Infrared spectroscopy in KNbO3through the successive ferroelectric phase transitions. Journal of Physics C: Solid State Physics **17**, 483–514 (1984).
5. Zhong, W., Vanderbilt, D. & Rabe, K. M. First-principles theory of ferroelectric phase transitions for perovskites: The case of $BaTiO_3$. Phys. Rev. B **52**, 6301–6312 (1995).
6. Hukushima, K. & Nemoto, K. Exchange monte carlo method and application to spin glass simulations. J. Phys. Soc. Jpn. **65**, 1604–1608 (1996).
7. Katzgraber, H. G., Trebst, S., Huse, D. A. & Troyer, M. Feedback-optimized parallel tempering monte carlo. J. Stat. Mech: Theory Exp. **2006**, P03018–P03018 (2006).
8. Nosé, S. A molecular dynamics method for simulations in the canonical ensemble. Mol. Phys. **52**, 255–268 (1984).
9. Nosé, S. A unified formulation of the constant temperature molecular dynamics methods. J. Chem. Phys. **81**, 511–519 (1984).
10. Hoover, W. G. Canonical dynamics: Equilibrium phase-space distributions. Phys. Rev. A **31**, 1695–1697 (1985).
11. Chen, P., Zhao, H., Artyukhin, S. & Bellaiche, L. LINVARIANT. `https://github.com/PaulChern/LINVARIANT` (2019).
12. Mankowsky, R., von Hoegen, A., Först, M. & Cavalleri, A. Ultrafast reversal of the ferroelectric polarization. Phys. Rev. Lett. **118**, 197601 (2017).
13. Nova, T. F., Disa, A. S., Fechner, M. & Cavalleri, A. Metastable ferroelectricity in optically strained $SrTiO_3$. Science **364**, 1075–1079 (2019).
